# Changes to coral health and metabolic activity under oxygen deprivation

**DOI:** 10.7717/peerj.1956

**Published:** 2016-04-19

**Authors:** James W.A. Murphy, Robert H. Richmond

**Affiliations:** Kewalo Marine Laboratory, Pacific Biosciences Research Center, University of Hawaii at Manoa, Honolulu, HI, United States

**Keywords:** *Montipora capitata*, Anoxia, Hypoxia, Enzyme activity, Alanopine dehydrogenase, Strombine dehydrogenase, Ecological resilience, Coral metabolism, Corals, Hawaii

## Abstract

On Hawaiian reefs, the fast-growing, invasive algae *Gracilaria salicornia* overgrows coral heads, restricting water flow and light, thereby smothering corals. Field data shows hypoxic conditions (dissolved oxygen (DO_2_) < 2 mg/L) occurring underneath algal mats at night, and concurrent bleaching and partial tissue loss of shaded corals. To analyze the impact of nighttime oxygen-deprivation on coral health, this study evaluated changes in coral metabolism through the exposure of corals to chronic hypoxic conditions and subsequent analyses of lactate, octopine, alanopine, and strombine dehydrogenase activities, critical enzymes employed through anaerobic respiration. Following treatments, lactate and octopine dehydrogenase activities were found to have no significant response in activities with treatment and time. However, corals subjected to chronic nighttime hypoxia were found to exhibit significant increases in alanopine dehydrogenase activity after three days of exposure and strombine dehydrogenase activity starting after one overnight exposure cycle. These findings provide new insights into coral metabolic shifts in extremely low-oxygen environments and point to ADH and SDH assays as tools for quantifying the impact of hypoxia on coral health.

## Introduction

### Global coral health

Coral reefs are important cultural, ecological, and economic resources, providing critical marine habitats for many invertebrates, fish, and algae species (McClanahan, Polunin & Done, 2002; Hughes et al., 2003). Their complex structure provides marine life with food, suitable habitats for growth, and protection from predators, while also acting as natural barriers that buffer adjacent coastlines from coastal erosion (McManus, 1997; White, Vogt & Arin, 2000; Cesar, Burke & Pet-soede, 2003; Bishop et al., 2011). However, despite their value, coral reefs have been devastated by increased anthropogenic impacts and changing abiotic environmental factors that continue to overwhelm these ecosystems (McClanahan, Polunin & Done, 2002; Hughes et al., 2003; Fabricius, 2005; De’ath et al., 2012; Bahr, Jokiel & Rodgers, 2015).

Destructive fishing practices, terrestrial run-off and pollution, sewage effluent infusion, coupled with intensifying storms, regional warming events, and invasive species impacts, have led to major losses in scleractinian coral cover, fish abundance, and decreased ability of reefs to support local human populations (Stimson, Larned & Conklin, 2001; Fox et al., 2003; Fabricius, 2005; Dailer et al., 2010). These continued pressures have resulted in mass coral bleaching events, and have the ability to disrupt spawning patterns and trigger widespread coral mortality (Downs et al., 2002; Levitan et al., 2014; Bahr, Jokiel & Rodgers, 2015; Paxton et al., 2015). As a result, mass losses of coral cover and subsequent shifts in the ecosystem balance has the potential to further reduce the abundance and diversity of fish and invertebrate species (Jones et al., 2004).

Historically, instances of mass coral mortality in combination to altered environmental conditions have led to phase shifts in coral reef structure, wherein coral reefs have shifted from a coral to an algae dominated state (McCook, 1999; Stimson, Larned & Conklin, 2001). Although herbivores, such as urchins and fish, can potentially limit the expansion of algal cover (Westbrook et al., 2015), aggressive invasive algae and herbivore grazing preferences for native species have had a profound effect on coral reefs, especially in the Hawaiian Islands where such algae have been found to overgrow coral colonies and negatively impact coral health (McCook, Jompa & Diaz-Pulido, 2001; Stimson, Larned & Conklin, 2001; Smith et al., 2004). These impacts include algal overgrowth creating oxygen and solar radiation-poor environments for corals, which induce bleaching through photoinhibition and reduced photorespiration (Martinez, Smith & Richmond, 2012).

Due to their distribution and lack of motility, corals are vulnerable to hypoxia-inducing circumstances including algal overgrowth, eutrophication, and sedimentation (Martinez, Smith & Richmond, 2012). Studies have been conducted documenting the negative impacts of oxygen deprivation on coral health (Stimson, Larned & Conklin, 2001; Smith et al., 2006; Fabricius, 2011; Jokiel et al., 2014); however, these studies relied upon proxies analyzing long-term effects and percent coral mortality. As such, there is a need to further understand fine-scale impacts to coral health on a molecular level in order to monitor changes in health to reveal negative impacts prior to mortality. In order to address this need, in this study we have developed enzyme assays, which can be used to quickly quantify hypoxic metabolic stress in the coral *Montipora capitata*, a major reef-building coral in Hawaii.

**Figure 1:**
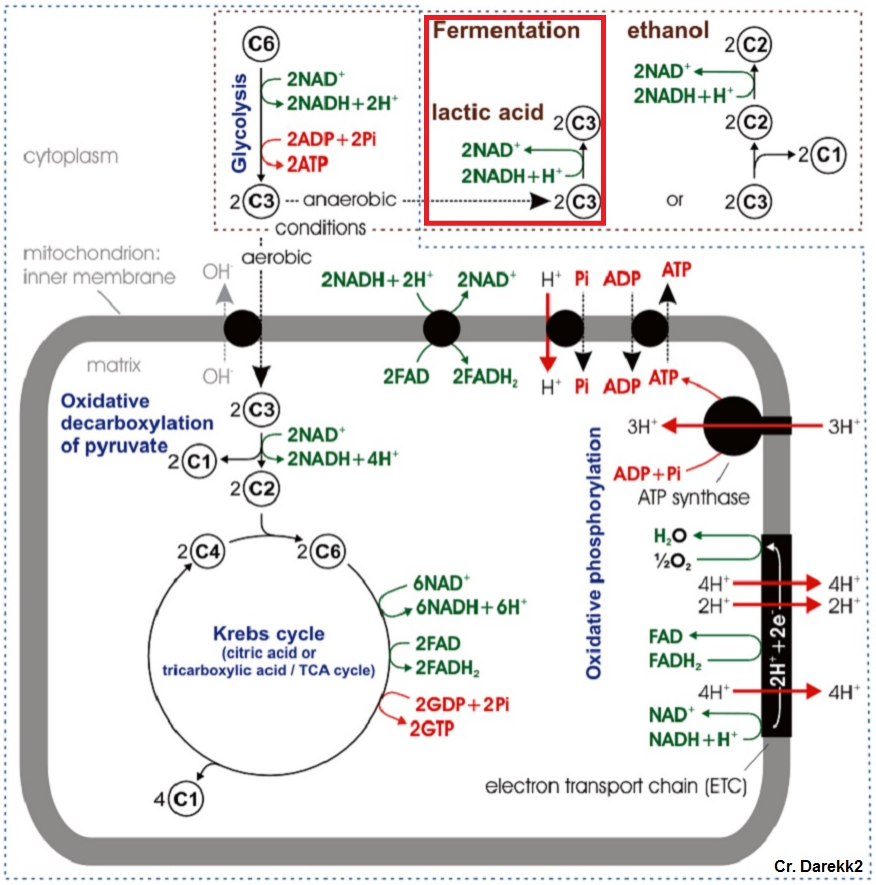
Typical cellular respiration pathway in eukaryotic cells. Red box denotes anaerobic respiratory pathway of interest.

**Figure 2:**
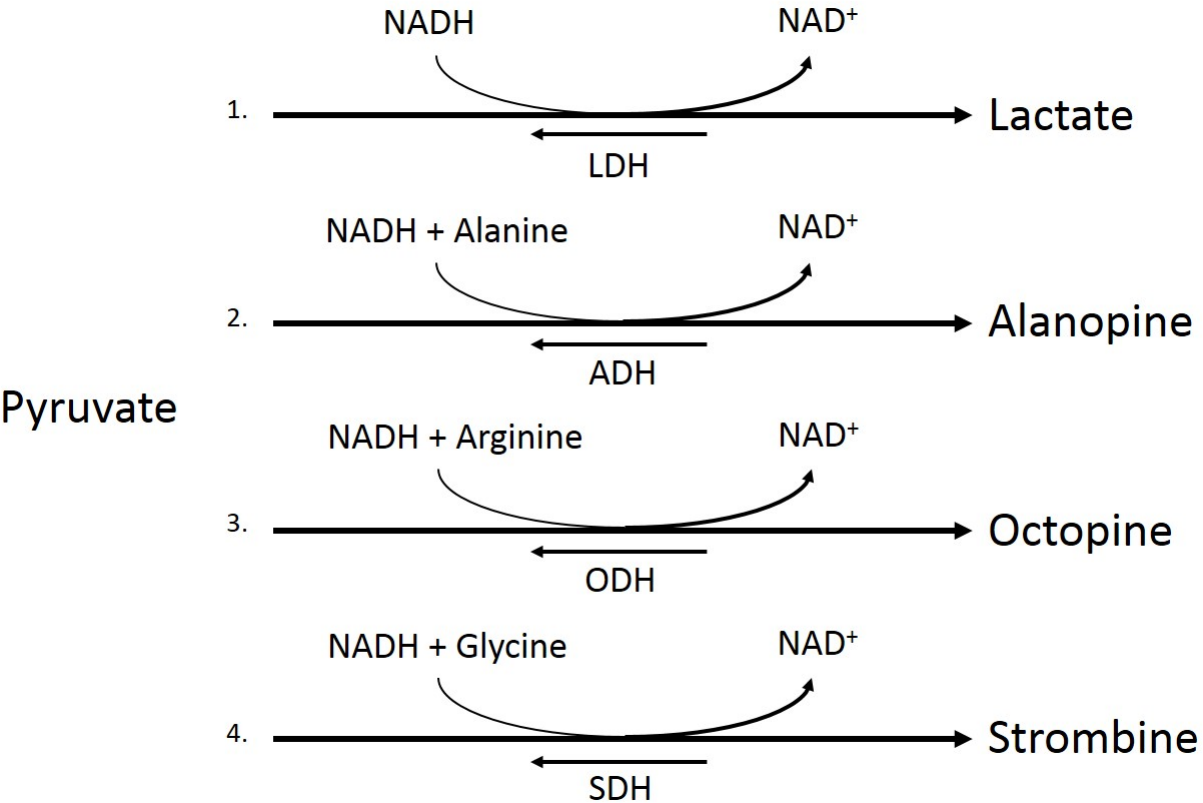
Pyruvate metabolism pathways. Conversion of pyruvate to lactate by lactate dehydrogenase (LDH, 1), pyruvate and alanine to alaopine by alanopine dehydrogenase (ADH, 2), pyruvate and arginine to octopine by octopine dehydrogenase (ODH, 3), and pyruvate and glycine to strombine by strombine dehydrogenase (SDH, 4).

### Coral metabolic biochemistry

Under low oxygen conditions, organisms shift from aerobic to anaerobic metabolism of glucose, allowing for the continuation of energy production under hypoxic to anoxic conditions (Fig. 1) (Nelson & Cox, 2008). Rather than entering the pyruvate dehydrogenase enzyme complex following glycolysis, pyruvate can be instead converted to lactate by the enzyme lactate dehydrogenase (LDH) (Fig. 2). While this reaction replenishes stores of NAD^+^, the required co-factor for catabolism of glucose molecules, it is accompanied by a significant decrease in net energy production from 38 moles to 2 moles of ATP per mole of glucose (Nelson & Cox, 2008). Marine organisms, specifically polychaetes and cnidarians, have been shown to survive periods of environmental hypoxia (dissolved oxygen (DO_2_) ≤ 2.0 mg/mL) for five or more days (Schottler, 1982; Martinez, Smith & Richmond, 2012). However, reduced capacity for ATP production under hypoxia puts marine invertebrates at a greater disadvantage in energy production if they must rely upon anaerobic respiration for longer periods of time (Livingstone, 1991). Many marine invertebrates, such as crustaceans and echinoderms, have been found to rely upon LDH and lactate production as a means of maintaining metabolism during oxygen stress (De Zwaan & Putzer, 1985). However, the activities of opine dehydrogenases (OpDH) have also been discovered in many marine invertebrate tissues (Plaxton & Storey, 1982; De Zwaan & Putzer, 1985; Livingstone, 1991; Sato et al., 1993; Lee, Lee & Pan, 2011). This enzyme suite, consisting of enzymes such as, alanopine dehydrogenase (ADH), octopine dehydrogenase (ODH), and strombine dehydrogenase (SDH) (Fig. 2), has been characterized in a wide variety of organisms and has been found, in most cases, to be the favored pathway in anaerobic respiration over LDH (Livingstone, 1991; Lee, Lee & Pan, 2011). Further, in many marine invertebrates, such as molluscs, polychaetes, cnidarians, and poriferans, OpDH activities have been found to be significantly higher than that of LDH (Sato et al., 1993).

Nevertheless, information regarding the activity and presence of these enzymes characterizing cellular anaerobic respiration is still limited. Though studies have been conducted analyzing the presence and production of LDH and OpDHs in the sea anemone *Diadumene leucolena*, little to no other published data exist describing the responses of LDH and OpDHs to oxygen deprivation in scleractinian corals (Ellington, 1977; Ellington, 1979). Therefore, we sought to determine the activity of enzymes associated with anaerobic respiration, ADH, ODH, SDH, and LDH, to better characterize anaerobic metabolism in corals under prolonged oxygen deprivation, such that these enzymes may serve as proxies for rapidly characterizing hypoxic stress in coral. Due to its wide-spread distribution and observations of invasive algal overgrowth and oxygen deprivation of *M. capitata* in Kaneohe Bay, Oahu, Hawaii, this species was chosen as the model coral for this study.

## Materials and Methods

### Collection

*Montipora capitata* nubbins were cultivated in open flow-through seawater tanks at the Kewalo Marine Laboratory (KML) under Department of Land and Natural Resources—Division of Aquatic Resources coral collection permit SAP 2012-6 (Oahu, HI, USA). The KML seawater system is fed by unfiltered seawater from an intake pipe 300 m offshore in 10 m deep water. Large coral colonies were fragmented into nubbins approximately 20 cm^3^ in size (*n* = 157) and attached to a substrate using Gorilla Glue (Cincinnati, OH, USA). These mounted nubbins were then suspended vertically in tanks for two weeks prior to any sampling in order to allow recuperation from stress resulting from manipulation. Nubbins were inspected and cleaned biweekly of sediment and algae.

### Hypoxic exposure

Following recovery, *M. capitata* nubbins were cut from their substrate and randomly assigned to a reference (${n}_{\mathrm{tank}}=5$), exposure control (${n}_{\mathrm{control}}=30$), or treatment (${n}_{\mathrm{treatment}}=30$) group. Reference nubbins were left on their substrate to account for stress resulting from the exposure setup. All other nubbins were placed in 250 mL beakers with 200 mL of seawater. Treatment beakers were bubbled with nitrogen gas (GasPro, Oahu, HI, USA) to remove oxygen from the seawater, while control beakers were bubbled with air using Tetra Whisper 20 Gallon air bubblers (Blacksburg, VA, USA). Gas bubbling was performed continuously during exposures. Hanna (HI 9828; Woonsocket, RI, USA) Multiparameter probes and YSI (6820-C-M; Yellow Springs, OH, USA) instruments were used to measure dissolved oxygen (ppm, % saturation, and mg/L), pH, and temperature at the start and finish of 12-hour bubbling periods. After 12 h of bubbling, beakers were returned to the open flow through tank and oxygen levels restored in order to mimic field-observed cycles of oxygen deprivation. This cycle of bubbling and ‘recuperation’ persisted for a maximum of 5 days and was conducted to simulate smothering conditions observed by Martinez, Smith & Richmond (2012).

**Figure 3:**
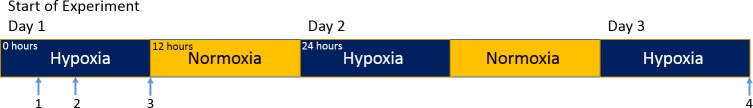
Sampling scheme displaying collections over treatment period (blue signifying overnight bubbling and yellow, the return to normal oxygen levels during the day). Arrow 1 corresponds to the ‘3 h’ collection time point, 2 to ‘6 h,’ 3 to ‘1 day,’ and 4 to ‘3 days.’ ‘Day 5’ of bubbling was omitted, as tissue loss rendered those samples unsuitable for analysis.

Beakers were sealed with Parafilm (Neenah, WI, USA) and fume hood sashes in which beakers were set up were lowered and covered with foil to block all light from entering during the treatments to inhibit the production of oxygen through photosynthesis by the symbiotic zooxanthellae. To further mimic field conditions, treatments began at sunset and concluded before sunrise. Following 3 and 6-hour and 1, 3, and 5-day bubbling cycles, coral nubbins were collected from their respective beakers (${n}_{\mathrm{treatment}}=5$ and ${n}_{\mathrm{control}}=5$ per time period; Fig. 3), gently and rapidly blotted dry, placed in 50 mL Falcon tubes, flash frozen with liquid nitrogen, and immediately transferred to a VWR 5656 −80 °C freezer (Radnor, PA, USA).

### Tissue preparation of S9 subcellular fractions

Liquid nitrogen was used to cool ceramic mortars and pestles, and pliers before crushing of coral nubbins. Pliers were used to fragment corals into smaller pieces before grinding them into a fine powder with a chilled mortar and pestle. Approximately 500 mg of crushed coral were transferred to 2 mL microcentrifuge tubes. Samples were homogenized on ice with 700 µL of homogenizing buffer (49,500 µL 0.01 M Tris–HCl pH 8.0, 500 µL dimethyl sulfoxide, 8.7 mg phenylmethylsulfonyl fluoride) for 60 s using an Ultra-Turrax homogenizer. Homogenates were centrifuged at 2,000 rcf for 5 min at 4 °C using an Eppendorf Microcentrifuge 5415D (Hauppauge, NY, USA) to pellet coral skeletal fragments and zooxanthellae. The supernatant was transferred to new microcentrifuge tubes and spun at 10,000 rcf for 20 min at 4 °C. The resulting supernatant, representing the S9 post-mitochondrial fraction of coral protein, was then aliquoted and stored frozen at −80 °C to preserve enzyme integrity and stop biological activity until analyses were performed.

### Protein concentration quantification

Protein concentration for all extracted samples was determined using the bicinchoninic acid (BCA) assay method. Clear 96-well microplates (Greiner 65580x; Grenier Bio-One, Monroe, NC, USA) were loaded with known dilutions of bovine serum albumin (BSA) (1.0, 0.7, 0.6, 0.5, 0.4, 0.3, 0.2, and 0.1 mg/mL; 20 uL per well) as standards, and unknown coral samples diluted 1:5 in milliQ water (20 uL S9 fraction with 80 uL water; 20 uL per well) in triplicate wells. The developing reagent (20,000 µL BCA solution, 400 µL ${\mathrm{Cu}}^{2+}{\mathrm{SO}}_{4}$) was made fresh prior to adding 200 uL per well. Plates were thoroughly mixed and then incubated at 37 °C for 30 min inside a SpectraMax M5 Multi-Mode Microplate Reader (Molecular Devices, Sunnyvale, CA, USA). Samples were then read using SoftMax Pro 5.4 software (Molecular Devices, Sunnyvale, CA, USA) at *λ* = 562 nm and protein concentrations calculated from the generated standard curve. For these assay purposes, all samples were diluted to 1 mg/mL for assay use and samples were re-extracted if protein concentration fell below the minimum threshold of 1 mg/mL.

### Chemicals

The following chemicals (analytical grade or higher) were purchased Sigma-Aldrich Chemical (St. Louis, MO, USA): L-alanine, L-arginine, glycine, and NADH. All other chemicals or reagents were purchased from VWR Scientific (Batavia, IL, USA) and were analytical grade or higher.

### Enzyme kinetic assays

Activity of LDH, ADH, SDH, and ODH was determined by monitoring the loss of absorbance due to the oxidation of NADH to NAD+ as previously described by Fields et al. (1980), Fields & Hochachka (1981), Lee, Lee & Pan (2011), Zewe & Fromm (1965a) and Zewe & Fromm (1965b). On ice, Greiner optically-clear 96-well plates were loaded with sodium pyruvate, Tris–HCl buffer, L-alanine/L-arginine/glycine, and sample S9 protein fraction diluted to 1 mg/mL in triplicate, and then incubated for 3 min at 28 °C (LDH assays employed sodium pyruvate and substituted L-alanine/L-arginine/glycine volumes with buffer). The reaction was then initiated with the addition of NADH. The plate was then mixed and read at *λ* = 340 nm for 30 min at 28 °C (30 s read intervals) (Horecker & Kornberg, 1948).

Activity units were defined as nanomoles of NADH oxidized per minute per mg protein (nmols/min/mg prot). The assay conditions of opine dehydrogenases were 100 mM sodium pyruvate, 100 mM Tris–HCl (pH 7.2), 1 mM NADH, 200 mM L-alanine/glycine/L-arginine, and 10 µg S9 protein fraction, in a final reaction volume of 100 µL. LDH activity was determined in the same reaction mixture with the omission of amino acids and a corresponding increased volume of buffer. Assay controls were carried out by the substitution of substrates with buffer to account for the endogenous levels of NADH degradation within the coral sample. Background activity levels were subtracted from observed sample activities.

## Results

### Visual observations

Though not the focus of this study, qualitative visual observations of coral health were tracked in order to monitor any physical changes in coral health during hypoxia exposure and draw comparisons between observations in the field.

Visual observations of tank controls found no observable physical changes over time, as tissue remained dark orange-brown in color. Polyps were regularly extended and appeared large and healthy with no mesentery filament extrusion. Likewise, air-bubbled exposure controls showed no physical changes, including response to bubbling, over the duration of testing. Polyps remained brown in color and were fully extended. No tissue loss was observed in any of the tank or exposure controls. However, hypoxia treatment corals displayed many physical changes, worsening with the duration of exposures.

No physical signs of stress were seen in corals immediately following the first night (12 h) of nitrogen bubbling. However, at the start of the second night of bubbling, coral samples displayed slight bleaching and color loss. Treatment polyps remained contracted during the duration of exposures. Continued treatment resulted in increased bleaching and tissue loss on coral nubbins, but no coral nubbin deaths. By the fifth cycle of testing, coral nubbins under hypoxic conditions were almost completely bleached or had lost all tissue, fitting observations of field-observed corals (Martinez, Smith & Richmond, 2012). Remaining polyps were dark brown and appeared shriveled. Due to the low amount of remaining tissue, day 5 samples were unsuitable for processing.

To note, pH and temperature did not significantly vary between treatment, control, and reference tank conditions.

### Statistical analyses

Statistical analyses were performed using the program Prism 5 (GraphPad Software, La Jolla, CA, USA). Through a two-way analysis-of-variance (ANOVA) using Bonferroni post-hoc tests, the differences in enzyme activity over the 4 treatment times (3 and 6 h and 1 and 3 days) and 3 treatment types (treatment, air-bubbled controls, and tank controls) were determined. This test investigated the significance of differences between treatment times, treatment types, and differences in the interaction between treatment times and types, within each enzyme activity assay. This interaction between time and type describes whether time and type had a significant combinatory effect on changes in enzyme activity.

### Enzyme activity

**Figure 4:**
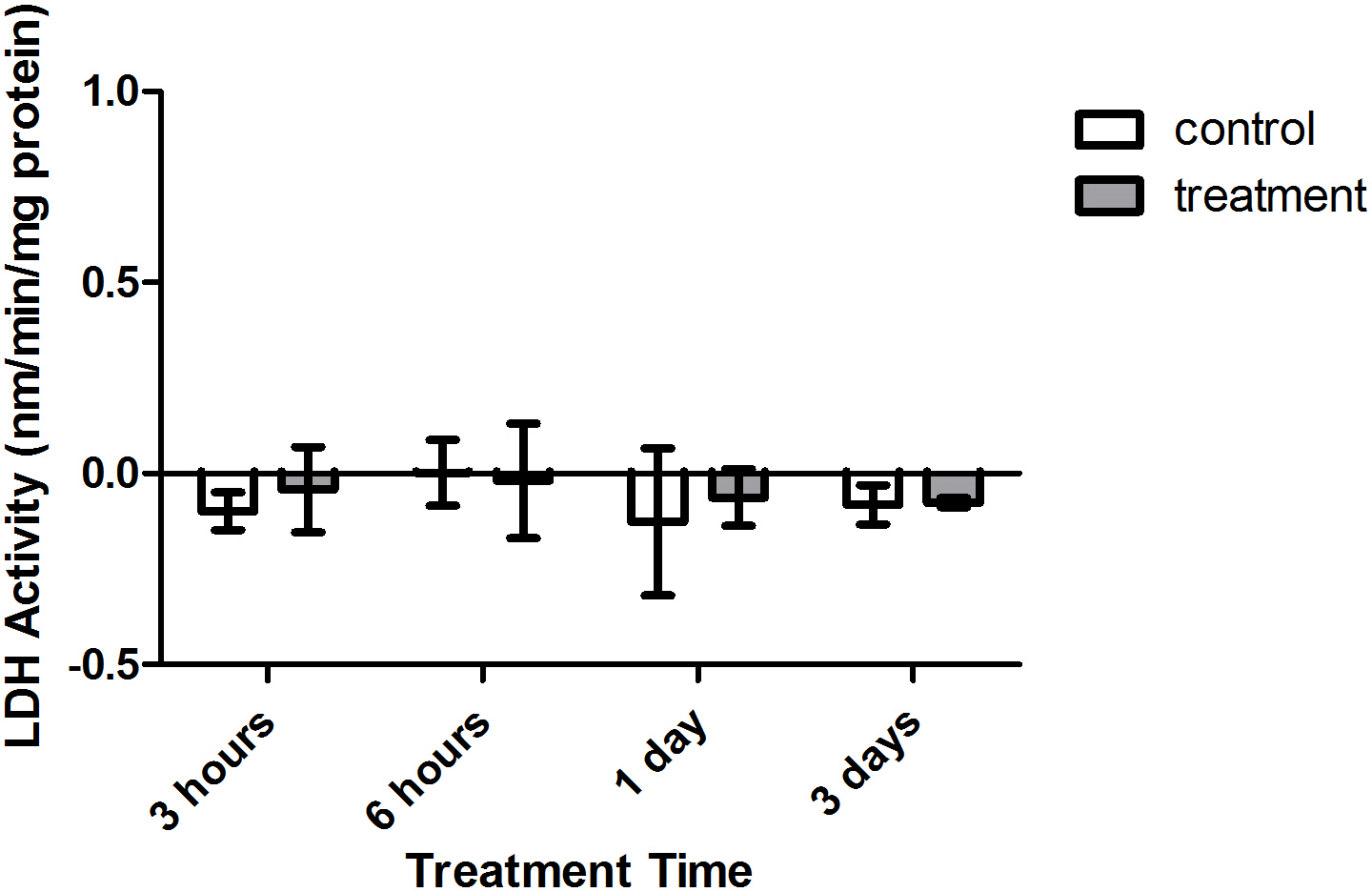
Lactate dehydrogenase (LDH) activity (nmols/min/mg pro) versus treatment time and type (3, 6 h and 1, 3 days). Bars represent mean ± SD.

**Figure 5:**
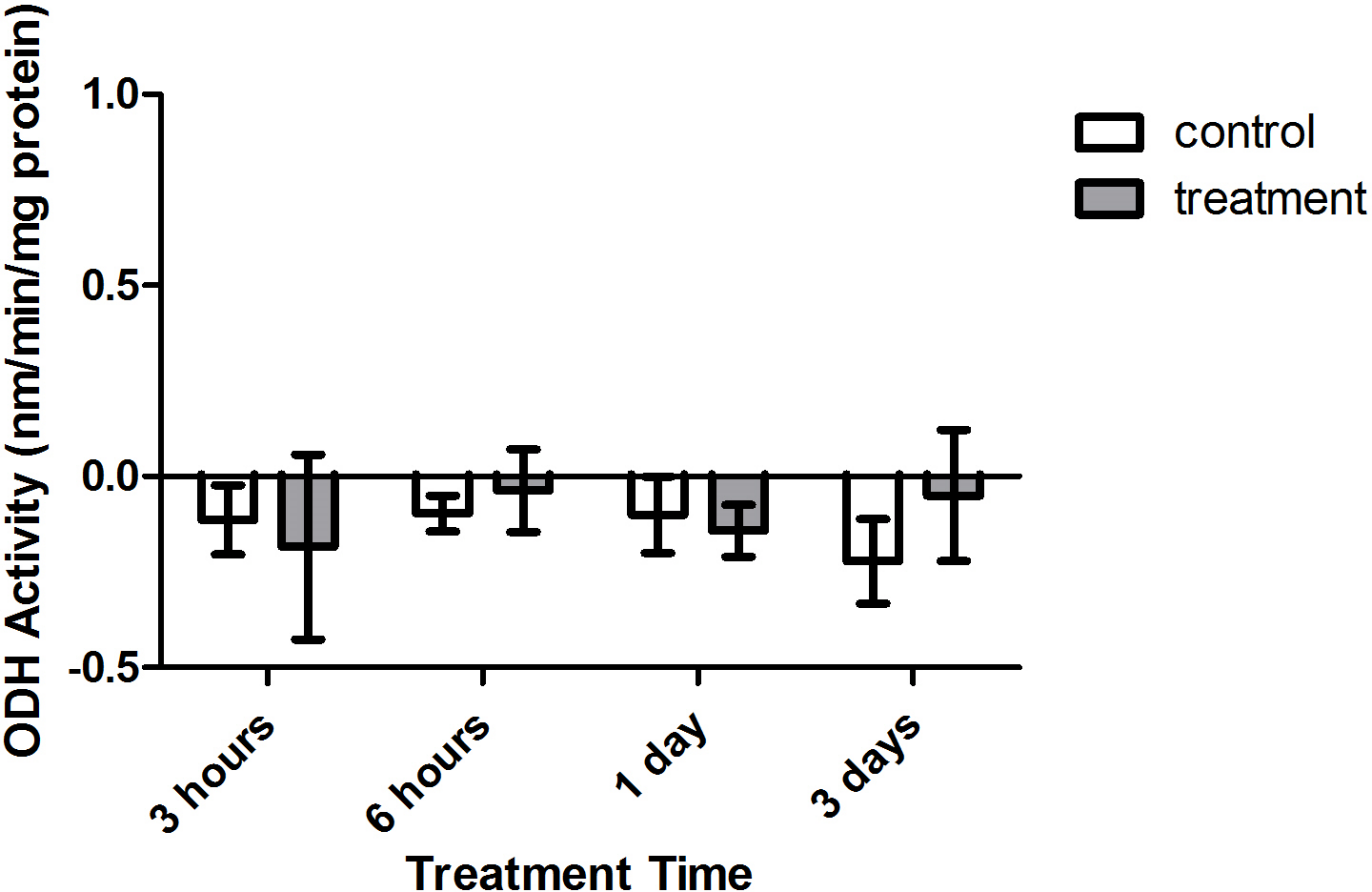
Octopine dehydrogenase (ODH) activity (nmols/min/mg prot) versus treatment time and type (3, 6 h and 1, 3 days). Bars represent mean ± SD.

Enzyme kinetic assays detected little to no LDH or ODH activity in coral samples (Figs. 4 and 5, respectively). Although several samples across treatment type and time displayed small positive values for enzyme activity, these were not significant increases in activity over control values (*p* > 0.05, CI = 95%).

**Figure 6:**
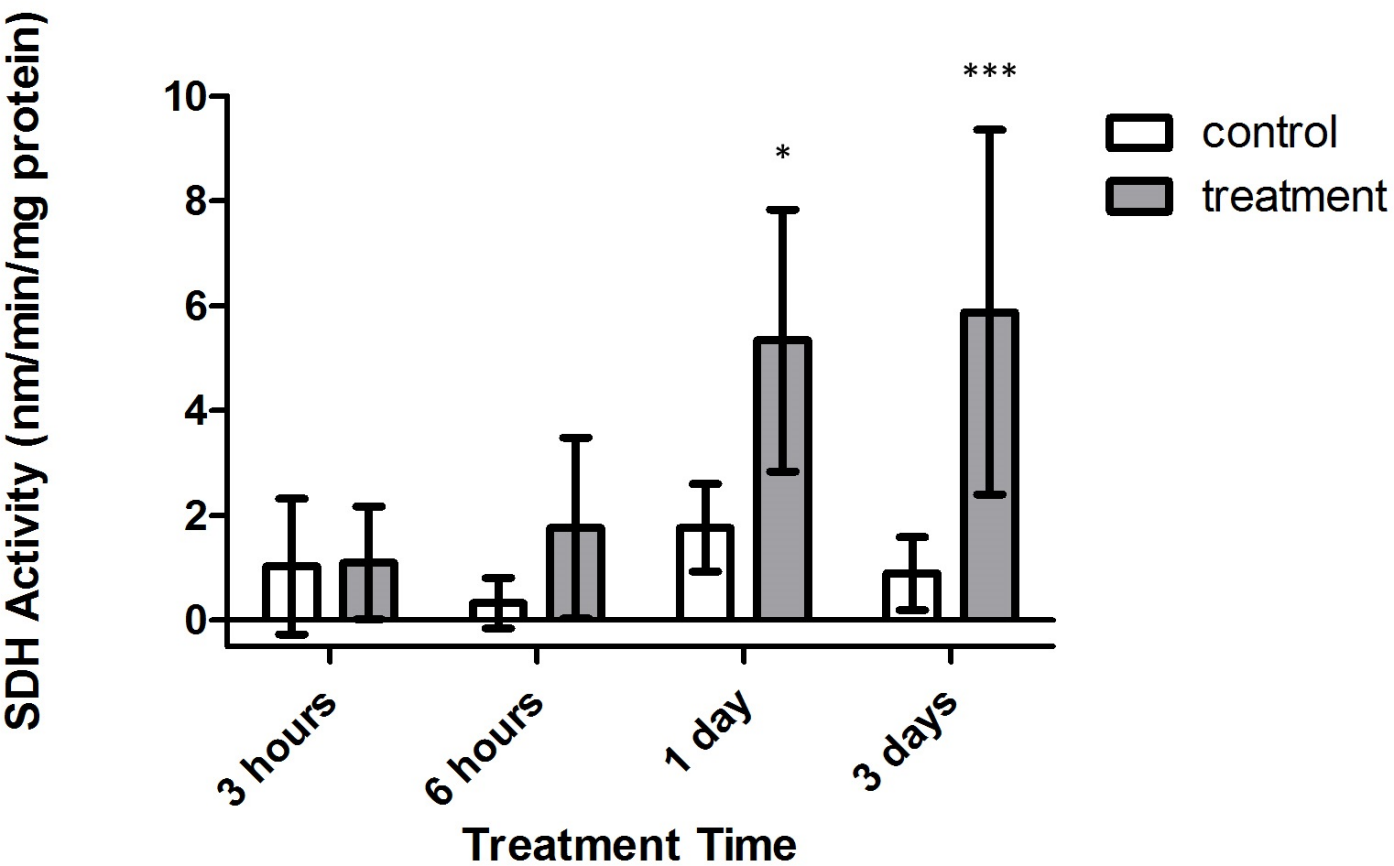
Strombine dehydrogenase (SDH) activity (nmols/min/mg prot) versus treatment time and type (3, 6 h and 1, 3 days). Bars represent mean ± SD. Treatments with significantly higher SDH activity than controls are denoted in treatments marked with asterisks (^∗^, *p* < 0.05; ^∗∗∗^, *p* < 0.001)

Conversely, significant increases in SDH activity were found to occur within treatment corals, with samples from both 1 and 3 days expressing significantly higher activity versus control corals (Fig. 6, *p* < 0.05 and *p* < 0.001, CI = 95%, respectively). Further, when investigating the impact of oxygen deprivation, exposure time, and the interaction of both factors on the metabolic activity of these samples, it was found that SDH activity varied significantly with respect to treatment type (*F* = 65.57, *p* < 0.0001), treatment duration (*F* = 4.944, *p* = 0.0082), and interaction between treatment duration and type (*F* = 3.039, *p* = 0.0485). Importantly, control and tank samples displayed no significant differences over 3 h, 6 h, 1 day, and 3 day times sets (*p* > 0.05, CI = 95%), indicating that bubbling within small chambers had no effect on target enzyme activity.

**Figure 7:**
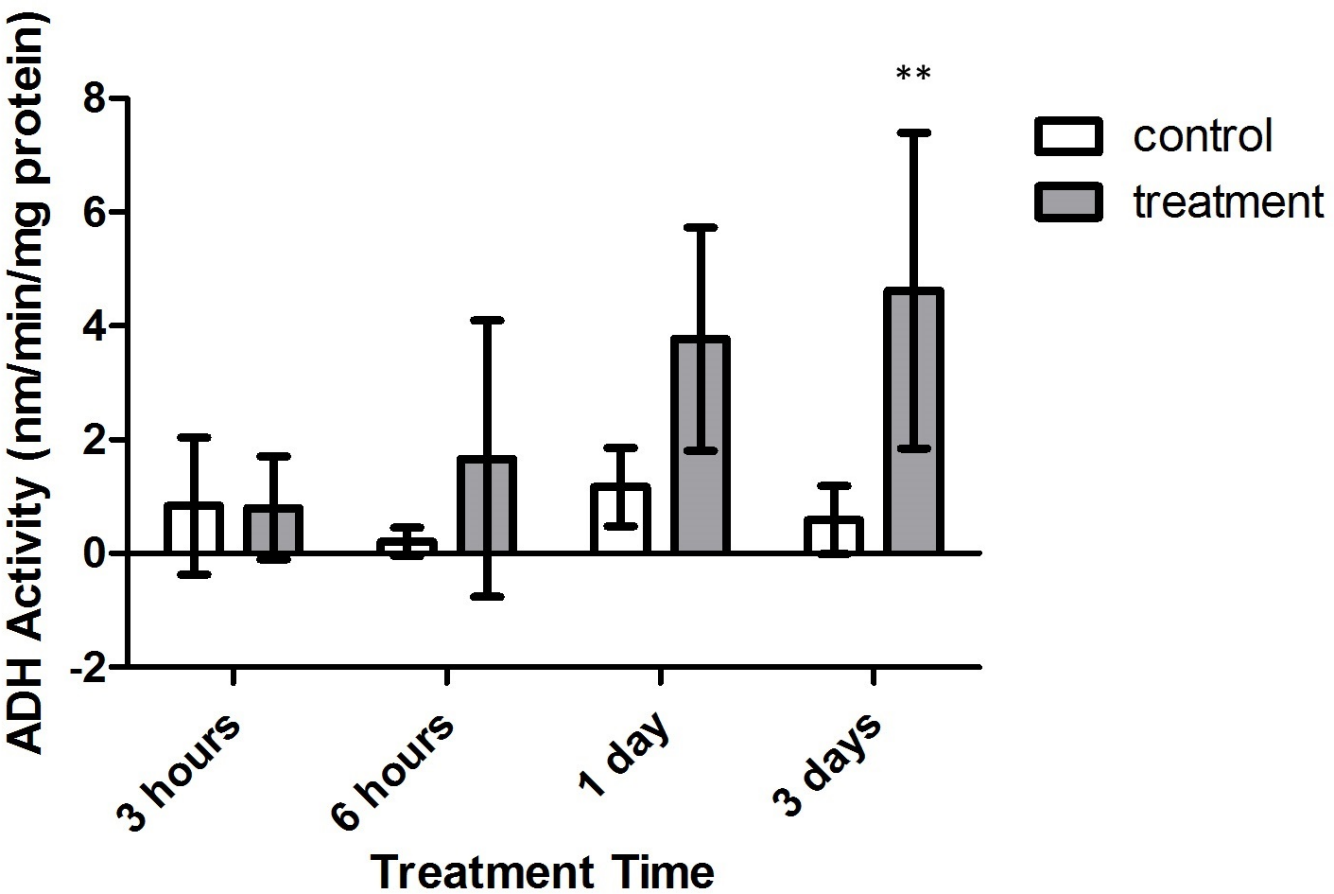
Alanopine dehydrogenase (ADH) activity (nmols/min/mg prot) versus treatment time and type (3, 6 h and 1, 3 days). Bars represent mean ± SD. The 3 day treatment with significantly higher ADH activity than controls is denoted by an asterisk (^∗∗^, *p* < 0.01).

Alanopine dehydrogenase (ADH) activity reflected similar findings as in SDH activity assays, where activity varied significantly with treatment type (*F* = 26.30, *p* = 0.0009) and time (*F* = 3.162, *p* = 0.0430). However, the combinatory effect of oxygen deprivation over time had no significant effect on ADH activity (*F* = 2.545, *p* = 0.0799). As in SDH activity results, ADH activity did not significantly differ between air-bubbled controls and tank references (*p* > 0.05, CI = 95%). Yet, as seen in Fig. 7, differences in ADH activity significantly differed between both control groups and treatment corals after 3 days of treatment (*p* < 0.01, CI = 95%). Overlap with control activity values resulted in no significant variation in ADH activity during 3 h, 6 h, and 1 day exposure periods (*p* > 0.05, CI = 95%).

## Discussion

Increased activity of ADH and SDH enzymes in *M. capitata* exposed to prolonged durations of hypoxic conditions (>12 h) mimic other recorded increases in gastropod species (Dando, 1981; Fields & Hochachka, 1981; Plaxton & Storey, 1982; Eberlee, Storey & Storey, 1983; Lee, Lee & Pan, 2011), as well as the sea anemone *Diadumene leucolena* (Ellington, 1979). Though sequential significant increases in SDH and ADH activity were not found with increasing time intervals, treatment values in Figs. 6 and 7 demonstrate general trends of increasing activity with increasing duration of treatment. These results suggest that under prolonged intervals of anoxia, *M. capitata* becomes increasingly dependent on the activity of ADH and SDH to mediate anaerobic metabolism.

While ODH and LDH activities have been monitored in the sea anemone *Bunodosoma cavernata* and various ctenophores and poriferans, their activity was barely detectable in *M. capitata* during this study (Livingstone, 1991; Sato et al., 1993). Although it is well documented that ODH and LDH are important in the processing of glycolytic products under anaerobic conditions (Sato et al., 1993; Lee, Lee & Pan, 2011), our lack of evidence does not mean that ODH and LDH do not exist in coral. The longest analyzed hypoxic treatment employed in this study was 3 days. However, it has been found that under chronic exposure to multiple weeks of hypoxia, other pathways for anaerobic respiration can be activated, becoming a significant pathway for metabolic turn over (De Zwaan & Putzer, 1985). Additional studies propose that ADH and SDH act as the primary and anaerobic metabolic responses pathways during hypoxia, while ODH is important for chronic hypoxia exposure (De Zwaan & Putzer, 1985).

The significant positive relationship between exposure length and enzyme activity of ADH and SDH in *M. capitata* supports the implementation of these enzymes as biomarkers for rapid analysis of hypoxia-induced stress in corals. However, further research is necessary to provide additional evidence to confirm the presence of these enzymes through Western Immunoblotting or other molecular techniques. Furthermore, future studies monitoring the effect of long-term oxygen deprivation are required to fully understand variation between acute and chronic coral metabolic responses. Additionally, investigation of endogenous substrate and co-factor concentrations for these target enzymes within tissues through further colorimetric assays can help elucidate changes in the overall pathways and provide a better understanding of homeostatic health and subtler molecular alterations resulting from hypoxic exposure.

Management strategies have been employed to mitigate the impacts from stressors, such as overgrowth by invasive algae (Kittinger et al., 2013; Westbrook et al., 2015). However, these efforts occur once a stressor has already inflicted a significant amount of damage to a coral reef (Stimson, Larned & Conklin, 2001; Conklin & Smith, 2005; Wolanski, Martinez & Richmond, 2009). Through the expansion of the cache of available biomarkers, we can better characterize stress levels in coral before bleaching and tissue loss/death occur. With this knowledge, we can actively sample corals that are found in environments that could be considered ‘less than optimal’ and specifically analyze the expression and activity of enzymes that should be up-regulated; ADH and SDH in low-oxygen environments, for example. Through this process, it would then be possible to proactively target coral reefs that are under ‘stress loads of concern,’ subsequently addressing the specific factor or factors eliciting the response. If explicitly looking to aid corals affected by invasive algal overgrowth, by understanding how severely increased the response of these enzymes are across a reef or several reefs, environmental managers will then be more efficiently able to prioritize efforts for the removal of invasive algae and/or the implementation of preventative measures, such as seeding reefs with herbivores, to improve environmental quality.

Calls for such integration and collaboration between research science and coral reef management programs have been addressed in various papers (Hughes et al., 2003; West & Salm, 2003; Aswani et al., 2015; Westbrook et al., 2015) and have led to successful reef management campaigns, such as that by The Nature Conservancy of Hawaii through their ‘Super Sucker’ and urchin bio-control programs. Further, many new studies leave room for the expansion of the analyses of coral response to factors such as hypoxia through the employment of newly described *Acropora* proteome data (Dunlap et al., 2013). This will allow for the targeting of other enzymes of interest, such as hypoxia-inducible factors (HIF) 1 and 2, for use in rapid coral stress response analyses, complementing current findings, and expanding upon available biomarkers for use in assessing coral health in reef management programs.

## Conclusions

Our findings indicate that, *M. capitata* increasingly relies upon ADH and SDH for anaerobic catabolism under low-oxygen conditions. This demonstrates that prolonged or repeated exposure to anoxia drives corals to rely upon less efficient anaerobic means of energy production. Under extended periods of low-oxygen exposure this may lead to energy deficits, which could result in increased susceptibility of corals to acute stress events, further leading to bleaching and tissue loss. Future efforts will focus on the application of these methods toward the analysis of oxygen deprivation on corals of different genera, elucidating possible variations in enzymatic stress response. These findings point to ADH and SDH activity assays as suitable biomarkers for rapid analyses of hypoxia-induced stress in corals with use in future analyses of environmental impacts on corals and coral reefs.

## Supplemental Information

10.7717/peerj.1956/supp-1 Data S1 Alanopine dehydrogenase and strombine dehydrogenase raw data Dataset includes values for control, treatment, and reference corals (labeled ‘tank’) for both alanopine and strombine dehydrogenase activity (units: nmols/min/mg protein).

## Additional Information and Declarations

### Field Study Permissions

The following information was supplied relating to field study approvals (i.e., approving body and any reference numbers): Department of Land and Natural Resources—Division 106 of Aquatic Resources coral collection permit SAP 2012-6 (Oahu, HI, USA).

### Data Availability

The following information was supplied regarding data availability: Raw data consists of enzyme activity values and has been supplied as Data S1.
